# Comparison of Westergren and Automated Erythrocyte Sedimentation Rate Measurements in Dogs and Cats

**DOI:** 10.3390/ani16152298

**Published:** 2026-07-24

**Authors:** Marisa Masucci, Alessandra Caprì, Alessandra Cartocci, Daniela Diamanti, Giulia Donato, Nicola Maria Iannelli, Carolina Pieroni, Flavia Rosace, Maria Grazia Pennisi

**Affiliations:** 1 Department of Veterinary Science, University of Messina, 98168 Messina, ME, Italy; marisa.masucci@unime.it (M.M.); nicolamaria.iannelli@unime.it (N.M.I.); flavia.rosace@studenti.unime.it (F.R.); 2 ASC “I Periodeuti”, 89122 Reggio Calabria, RC, Italy; alessandracapri@hotmail.com (A.C.); mariagrazia.pennisi@unime.it (M.G.P.); 3 Clinica Veterinaria Camagna, VetPartners, 89124 Reggio Calabria, RC, Italy; 4 Department of Molecular and Developmental Medicine, University of Siena, 53100 Siena, SI, Italy; alessandra.cartocci@dbm.unisi.it; 5 DIESSE—Diagnostica Senese S.p.A. Società Benefit, 53035 Monteriggioni, SI, Italy; danieladiamanti@diesse.it (D.D.); carolinapieroni@diesse.it (C.P.)

**Keywords:** ESR, Westergren method, MINI-PET, point of care test, reference range, EDTA blood, citrate blood, dog, cat, method comparison

## Abstract

The erythrocyte sedimentation rate (ESR) is a routine marker of inflammation in human medicine, and it is influenced by erythrocyte features and the level of some plasma proteins. The Westergren method is the reference technique in humans; however, this manual technique has been scarcely used in animals and has not been validated. An automated veterinary in-clinic device (MINI-PET) is able to measure ESR on EDTA-blood tubes. This study aimed to evaluate the comparability of ESR values measured with two tests in cats and dogs admitted to a veterinary clinic and in anemic individuals. Analytical comparability and decision limits were calculated in both species and clinical groups. Comparisons were also made between the two species. The two ESR methods had different decision limits in the investigated population of dogs and cats, and a method-specific interpretation of their results is suggested. Healthy cats had significantly higher values than healthy dogs using both the Westergren and MINI-PET methods. By both methods, anemic individuals had higher ESR values than the matched subgroups with erythrogram values within the reference range, probably due to concurrent inflammation in most anemic animals studied. In general, ESR measures should be evaluated in conjunction with erythrogram values.

## Simple Summary

The erythrocyte sedimentation rate (ESR) is a physical phenomenon measurable (mm/h) in anticoagulated blood samples, and it has been used as a routine marker of inflammation in humans for over a century. The Westergren method is the reference measurement technique in humans; however, this manual technique has been scarcely used in veterinary medicine and was not validated as an inflammatory marker. An automated veterinary in-clinic device (MINI-PET) was recently introduced to the market, and it is able to measure ESR on feline and canine EDTA-blood tubes. This study aimed to evaluate the comparability of ESR values measured with two tests in cats and dogs admitted to a veterinary clinic and in anemic individuals. Comparisons were also made between the two species. Analytical comparability and decision limits of the two methods were calculated in both species and the clinical groups. The two ESR methods had different decision limits in the studied dogs and cats, and a method-specific interpretation of their results is suggested. Cats had higher ESR decision limits compared to dogs. Anemia was associated with higher ESR values probably due to concurrent inflammation in most individuals; in general, ESR measures should be evaluated in conjunction with erythrogram values.

## 1. Introduction

**Figure 1:**
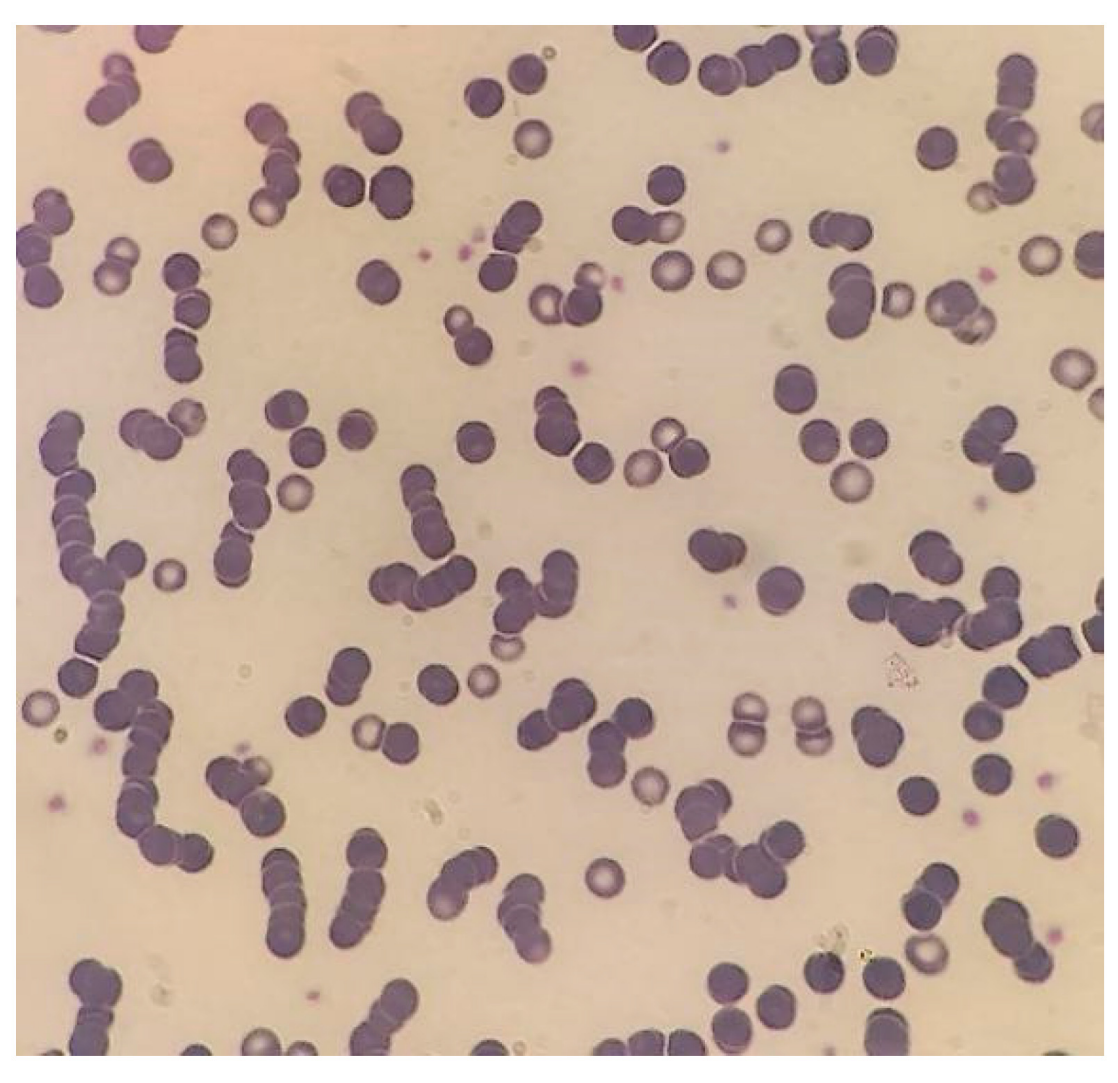
Rouleaux in a feline blood smear. May–Grünwald–Giemsa stain; 1000× magnification.

The erythrocyte sedimentation rate (ESR) is a century-old blood parameter based on the observation that the speed of sedimentation of red blood cells (RBCs) inside a test tube of anticoagulated blood varies in some physiological and pathological conditions [1,2]. The physical phenomenon of RBCs sedimentation due to the force of gravity is observed and measured (mm/h) in standardized transparent vertically set-up tubes. Sedimentation is preceded by aggregation of RBCs as “stack of coins” or “rouleaux” (Figure 1), and the pathomechanisms involved are still under debate [3]. 

There is a strong affinity among acute phase proteins, immunoglobulins, and other proteins with a positive electrical net charge (e.g., fibrinogen, α_2_-macroglobulin, IgM) and the negative surface RBC membrane. This phenomenon overcomes the electrostatic repulsion among erythrocytes and speeds up RBC sedimentation [2,3,4]. An increase in plasma proteins associated with inflammation induces faster erythrocyte sedimentation, and the ESR value is recognized as a non-specific marker of inflammation. Anemia and some size and shape changes in erythrocytes accelerate sedimentation [5,6,7,8,9,10,11,12]. However, a few erythrogram abnormalities are themselves caused by chronic inflammation (e.g., mild normocytic normochromic anemia) or concomitant with an acute phase reaction (e.g., associative or non-associative immune-mediated hemolytic anemia) [13,14,15]. The occurrence of anemia is taken into account in case of elevated ESR values; however, it does not change the role of ESR as a non-specific disease marker. 

The ESR is still a routine test in human medicine, and the Westergren method is the reference technique for the validation of ESR measures with new automated methods according to the International Council for Standardization in Hematology (ICSH) [2,4,16]. 

The ESR measured by the Westergren method has been scarcely used in canine and equine medicine, and it has not been validated as an inflammatory biomarker [4,17]. Recently, ESR studies have been performed on dogs and cats using a point-of-care (POC) automated device measuring ESR in EDTA blood test tubes (MINI-PET, DIESSE Diagnostica Senese S.p.A., Monteriggioni, SI, Italy) [4]. Some of these studies compared ESR values with various blood parameters, including established canine [18,19,20,21,22] and feline [12,23] positive and negative markers of inflammation. 

These studies support the fact that the ESR measured with MINI-PET is a promising marker of disease, particularly with respect to inflammation, in cats and dogs. However, there are some gaps in knowledge that should be filled. The influence of species-specific physiological (breed, sex, and age), reproductive (heat and pregnancy), and pathological (e.g., type and severity of inflammation, anemia) variables still needs to be investigated. 

The analytical comparability among Westergren and MINIPET methods—the two ESR measurement techniques currently available in cats and dogs—is of clinical relevance and has only been assessed in a single study including 60 healthy and sick cats [23].

The main aim of the present study was to evaluate the analytical comparability of ESR measurements obtained using MINIPET (ESR M-P) and Westergren (ESR W) assays in a population of cats and dogs admitted to a companion animal clinic. Comparisons were also made between the two species, taking into account the occurrence of anemia in the cats and dogs studied. Decision limits of the two methods in the studied species were calculated. We expect to yield new and actionable insights into the interpretation of ESR values in the case of data measured with different methods and in anemic animals.

## 2. Materials and Methods

### 2.1. Study Sites, Patient Enrollment, and Sampling Procedures

From June 2023 to May 2025, dogs and cats admitted to the Clinica Veterinaria Camagna-Vet Partners (Reggio Calabria, RC, Italy) were evaluated. Animals were presented for routine health checks, elective surgery, and various clinical problems. The study protocol was approved by the Ethics Committee of the Department of Veterinary Sciences of the University of Messina (12/2023 bis). Animal owners signed informed consent forms, which provided relevant information about the study protocol. Demographic data (i.e., breed, age, and sex) and baseline clinical information were recorded.

Dogs and cats aged ≥ 6 months were included, irrespective of sex, breed, weight, and reproductive and clinical status. The availability of 1 mL EDTA blood [used for automated ESR and complete blood count (CBC) evaluations], as well as 1 mL of blood to be placed in a sodium citrate Westergren-ESR test tube, was an additional inclusion criterion. In the case of patients admitted for surgery, blood collection was performed before the surgical procedure. Both EDTA and sodium citrate test tubes were visually inspected before performing the analytical procedures, and they were excluded in cases of hemolysis, lipemia, icterus, or clots. If additional blood testing was required for individual clinical reasons, a larger volume of blood was taken and processed as needed. In the case of anemic individuals, clinical data and, when available, values of the following acute phase reaction markers were considered in the subgroups of anemic animals: C-reactive protein (dogs), serum amyloid-A (cats), haptoglobin (cats), and serum protein electrophoresis fractions (dogs and cats).

### 2.2. Erythrocyte Sedimentation Rate (ESR) Measure

The ESR was measured by the manual Westergren reference technique and using an automated device (MINI-PET, DIESSE Diagnostica Senese S.p.A., Monteriggioni, SI, Italy). Both tests were performed within one hour of blood collection, with blood tubes stored at room temperature and away from light and heat sources.

#### 2.2.1. Westergren Method

One milliliter of blood was placed in the Westergren test tube containing 0.25 mL of 3.8% sodium citrate solution (FL MEDICAL Torreglia, Padua, PD, Italy) [2,24,25]. Westergren glass graduated ESR pipettes with internal plastic pistons (210 × 4.45 mm) and ESR racks were used (ESR pipettes, FL MEDICAL, Torreglia, Padua, PD, Italy). Citrate blood was resuspended by gently inverting the tube 10 times without forming bubbles immediately before inserting the ESR pipette. Blood suction was obtained by pulling the internal piston upwards, with an automatic stop when the blood column reached the “0” mark. Filled ESR pipettes were placed into an ESR rack and kept in a vertical position away from direct light and vibrations and at room temperature (18–25 °C) [25]. The result was read by visual inspection of the graduated tubes after 60’ at the interface between the plasma and the settled RBCs column, and it was recorded in mm/h. In the case of uneven settling (diphasic pattern of sedimentation), the sample was excluded from the study. 

#### 2.2.2. MINI-PET Assay

A MINI-PET analyzer (DIESSE Diagnostica Senese S.p.A., Monteriggioni, SI, Italy) was used to measure ESR in EDTA blood following the procedure provided by the manufacturer, with slight modifications. In detail, the EDTA blood was resuspended by gently inverting the tube 10 times before starting the test. Afterwards, the test tube was left upright for a few seconds so the blood could flow from the bottom of the tube cap, and the closed tube was inserted into one of the four slots of the device. After 14 min, the measure of ESR (expressed as mm/h) was displayed on the MINI-PET screen, and the result was recorded. When an error message appeared at the end of the procedure, the sample was excluded from the study. 

### 2.3. Complete Blood Count (CBC)

Complete blood count was performed in the in-house laboratory with the EDTA blood tube (FL MEDICAL Torreglia, Padua, PD, Italy) used to evaluate ESR M-P soon after the ESR measurement. Throughout this study, two laser hematology analyzers (ProCyteDx, IDEXX laboratories, Westbrook, ME, USA, or Futurlab DF 50, Futurlab Srl, Limena, Padua, PD, Italy) were used to perform the CBC. ProCyteDx was replaced by Futurlab DF 50 for strategic management decisions, and before replacement, a comparison of the diagnostic performance of the two blood cell counters was made. Regarding the parameters evaluated in this study, no significant discrepancies emerged in the erythrogram parameters. We used the specific red blood cell and reticulocyte counts, hematocrit, and hemoglobin concentration reference intervals of each hematology analyzer. Blood smears were prepared with fresh whole blood immediately upon collection of blood, stained with May–Grünwald–Giemsa stain (Merck KGaA, Darmstadt, Germany), and evaluated microscopically under oil immersion at 1000× magnification to confirm the results of automated analyzers and detect morphological abnormalities in blood cells. In anemic animals, data about leukocyte alteration compatible with inflammation (detection of neutrophil left shift, toxic neutrophils, or reactive lymphocytes and monocytes) were considered. 

Erythrogram data were evaluated, and diagnoses of anemia and erythrocytosis were made, respectively, when erythrocyte counts and hematocrit (HCT) or hemoglobin concentrations were below or above the reference range provided by the analyzer. The regenerative response was evaluated based on the reticulocyte count. Anemia severity was classified according to HCT values as mild (HCT ≥ 20% in cats, ≥30% in dogs), moderate (HCT 14–19% in cats, 20–29 in dogs), or severe (HCT ≤ 13% in cats, ≤19% in dogs) [26]. 

### 2.4. Selection Criteria of Healthy Cats and Dogs

Healthy cats [Westergren, *n* = 17 (9 males and 8 females, aged 6–108 months); MINI-PET, *n* = 27 (12 males and 15 females, aged 6–114 months)] and dogs (*n* = 25; 14 males and 11 females, aged 12–144 months) were selected based on history, physical examination, CBC, biochemical profile, and serological analysis. The biochemical profile performed in dogs included at least the following parameters: blood urea nitrogen (BUN), creatinine, glucose, alanine aminotransferase (ALT), alkaline phosphatase (ALP), amylase, lipase, total proteins, albumin, globulins, albumin/globulin ratio, and C-reactive protein. In cats, at least BUN, creatinine, glucose, ALT, ALP, total proteins, albumin, globulins, albumin/globulin ratio, and serum amyloid A were measured. Both dogs and cats tested negative for anti-*Leishmania infantum* antibodies, and cats were negative for anti-feline immunodeficiency virus antibodies and feline leukemia virus antigenemia.

The ESR M-P values of 10/27 healthy cats were from a previous study that we performed, with identical inclusion criteria and methods [12]. These ESR data were used exclusively to calculate the feline MINI-PET detection limit.

### 2.5. Statistical Analysis

The distribution of continuous variables was evaluated by the D’Agostino–Pearson omnibus test, and descriptive statistics were performed for the investigated variables.

The Wilcoxon matched-pair signed rank test was used to compare the values of ESR obtained with the Westergren method and the MINI-PET analyzer.

The Kruskal–Wallis test, followed by Dunn’s multiple comparison test, was applied to compare ESR values among the overall study population and two subgroups: animals with red blood cell count, hematocrit, and hemoglobin values within reference ranges and anemic animals.

Spearman’s rank correlation test was employed to assess the correlation between the Westergren method and the MINI-PET analyzer. The critical value of the correlation coefficient (r_s_) was established on the basis of the number of pairs of scores for each pair of parameters evaluated [27]. The strength of correlations, according to the r_s_ absolute value, was classified as follows: r_s_ = 0.80–1: very strong correlation; r_s_ = 0.60–0.79: strong correlation; r_s_ = 0.40–0.59: moderate correlation; r_s_ = 0.20–0.39: weak correlation; r_s_ = 0.10–0.19: very weak correlation [28].

Additionally, a nonparametric regression analysis (Passing-Bablok assay) was performed to compare the two analytical methods by analyzing systematic bias (constant and proportional, indicated by the intercept and the slope, respectively). The absence of a significant constant difference is indicated by the 95% confidence interval (CI) of the intercept including 0, while the absence of a significant proportional difference between the methods is indicated by the 95% CI of the slope including 1. 

A Bland–Altman graph was used to visually assess agreement between the two measurement methods, plotting the difference between paired measurements on the *Y*-axis against their average on the *X*-axis. The systematic bias (mean of all differences) and the limits of agreement (95% CI: mean difference ± 1.96 standard deviation of differences) show whether one method consistently differs from the other and within what range. A significant systematic error occurs when the value 0 is not within the 95% CI. When the differences between the ESR values obtained with the two methods were not normally distributed, the bias and the limits of agreement were estimated using a non-parametric method. In this case, bias was indicated as the median of the differences, and the limits of agreement were estimated using the 2.5th and 97.5th percentiles [29].

According to the American Society for Veterinary Clinical Pathology’s (ASVCP) reference interval guidelines, reference intervals were not calculated due to the small healthy animal sample size (<20 in the case of cat Westergren measures). Tables of ascending values measured with both methods, along with histograms and mean or median values in healthy dogs and cats, are reported in the Supplementary Materials (Tables S1 and S2; Figures S1 and S2) [30]. Since only an elevation of the ESR has clinical significance, we identified upper extremity outliers by Tukey’s interquartile fences and excluded them before considering the maximum ESR M-P and ESR W values measured in healthy dogs and cats as respective species decision limits [30].

The significance threshold was set at *p* < 0.05. Statistical analyses were conducted using R (version 4.5.1.) and GraphPad Prism (version 9.0.0) statistical software.

## 3. Results

### 3.1. Cat Demographic Data

One hundred and thirty-four cats were included in this study. Most cats (122; 91%) were domestic short-haired; the breed of 11 cats was not reported, and one cat was a Birman. Sixty cats (44.8%) were males, and 74 (55.2%) were females. The age ranged between 6 and 180 months (median: 12 months, 25th percentile: 8 months, 75th percentile: 72 months) in 114 cats, while the exact age of 20 adult cats was unknown. 

#### 3.1.1. Feline ESR Descriptive Statistics in Cats

**Figure 2:**
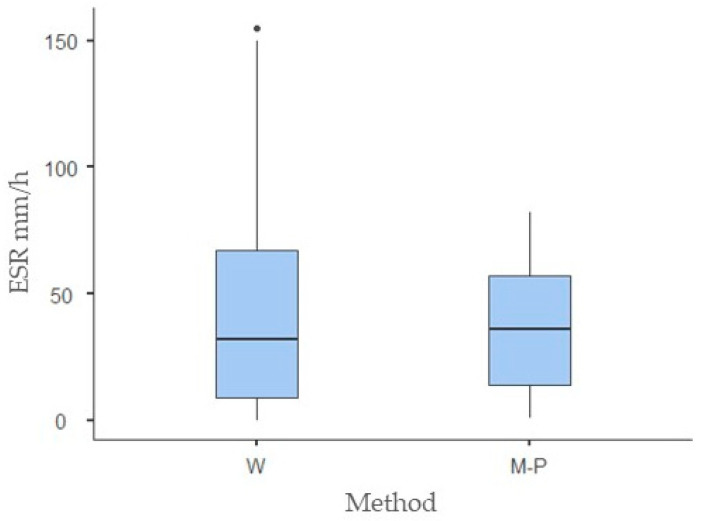
Box plot of ESR values obtained with the Westergren (W) method and MINI-PET (M-P) device in all cats examined.

The ESR M-P values did not differ significantly from ESR W measures (Table 1, Figure 2). 

**Table 1:** Descriptive statistics of the ESR values (mm/h) obtained with the Westergren (W) method and MINI-PET (M-P) device in all cats examined.

	W	M-P
Number of values	134	134
Minimum	0.00	1.00
25th percentile	9.00	14.00
Median	31.00	35.50
75th percentile	67.00	57.25
Maximum	155.00	82.00

**Figure 3:**
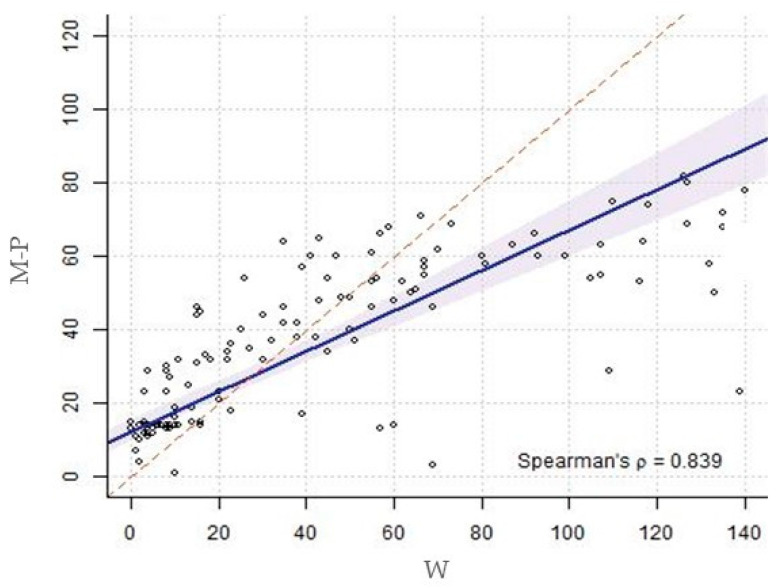
Passing–Bablok regression of ESR values (mm/h) obtained with the Westergren (W) method and MINI-PET (M-P) device in all cats examined.

A significant (*p* < 0.0001) and very strong positive correlation was detected between the ESR M-P and ESR W values (r*_s_* = 0.84). The intercept of the Passing–Bablok regression analysis was estimated at 12.17 (95% CI: 10.23–15.75), and the slope was at 0.55 (95% CI: 0.48–0.64) (Figure 3).

**Figure 4:**
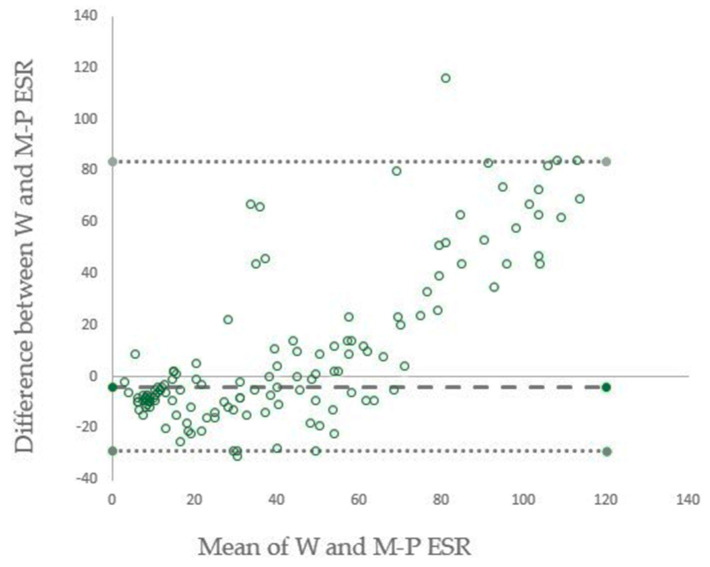
Bland–Altman plot of ESR values (mm/h) obtained with the Westergren (W) method and MINI-PET (M-P) device in all cats examined. Dashed line represents the bias (median of differences), and dotted lines indicate the upper and lower limits of agreement (2.5th and 97.5th percentiles).

The Bland–Altman analysis showed a bias of −4.5, with a 95% confidence interval ranging from −29 to 83.6 (Figure 4). 

The following decision limits were established in healthy cats after the exclusion of outliers (five ESR M-P and two ESR W): ESR W ≤ 15 mm/h and ESR M-P ≤ 25 mm/h.

#### 3.1.2. Feline ESR Descriptive Statistics in the Cat Subgroup with Erythrocyte Count, Hemoglobin, and Hematocrit Values Within Reference Ranges

**Figure 5:**
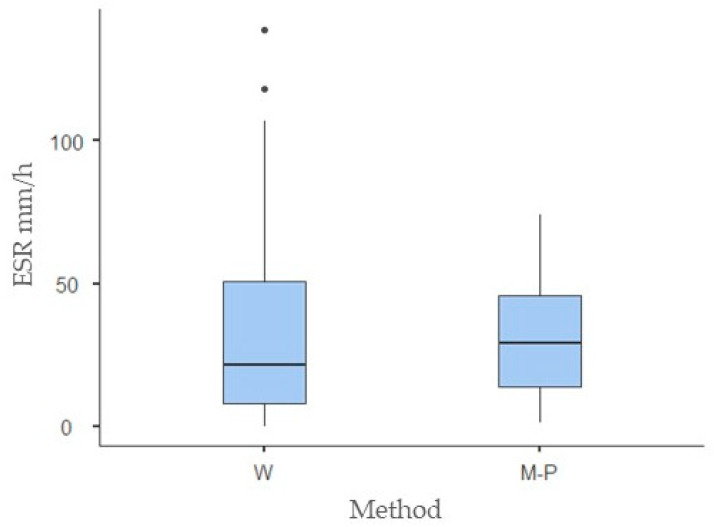
Box plot of ESR values obtained with the Westergren (W) method and MINI-PET (M-P) device in cats with erythrocyte count, hemoglobin, and hematocrit values within the reference range.

No significant differences were observed between ESR M-P and ESR W measures (Table 2, Figure 5).

**Table 2:** Descriptive statistics of the ESR values (mm/h) obtained with the Westergren (W) method and MINI-PET (M-P) device in cats with erythrocyte count, hemoglobin, and hematocrit values within reference ranges.

	W	M-P
Number of values	66	66
Minimum	0.00	1.00
25th percentile	7.50	14.00
Median	21.50	29.00
75th percentile	52.50	46.50
Maximum	139.00	74.00

**Figure 6:**
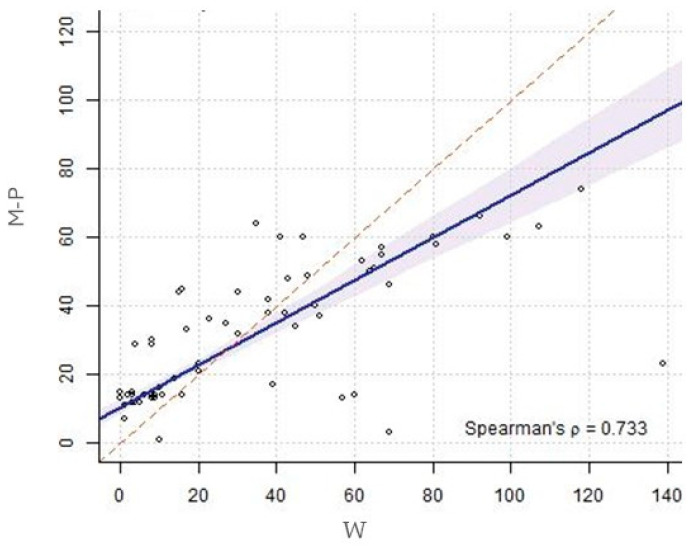
Passing–Bablok regression of ESR values (mm/h) obtained with the Westergren (W) method and MINI-PET (M-P) device in cats with erythrocyte count, hemoglobin, and hematocrit values within the reference range.

A significant (*p* < 0.0001), positive, and strong (r*_s_* = 0.73) correlation was detected between ESR M-P and ESR W tests. The intercept of the Passing–Bablok regression analysis was estimated at 19.52 (95% CI: 12.02–27.95), and the slope was at 0.44 (95% CI: 0.35–0.60) (Figure 6).

**Figure 7:**
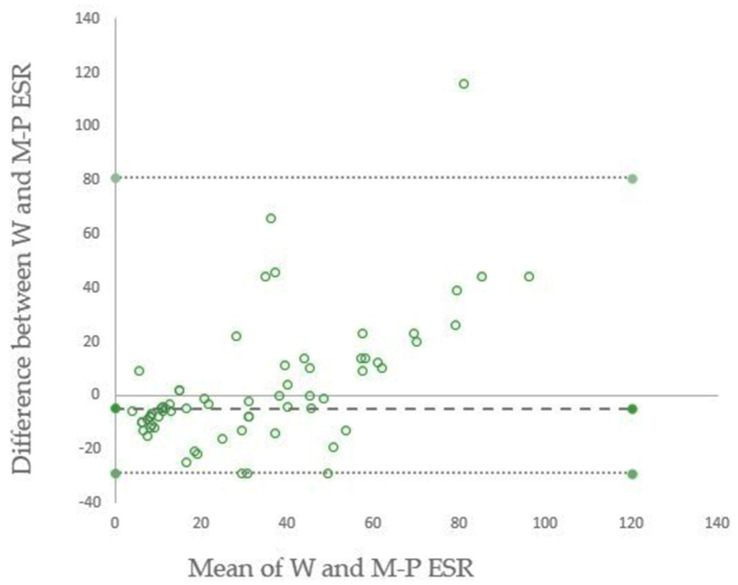
Bland–Altman plot of ESR values (mm/h) obtained with the Westergren (W) method and MINI-PET (M-P) device in cats with erythrocyte count, hemoglobin, and hematocrit values within reference ranges. Dashed line represents the bias (median of differences), and dotted lines indicate the upper and lower limits of agreement (2.5th and 97.5th percentiles).

The Bland–Altman analysis showed a bias of –5, with 95% limits of agreement ranging from −29 to 82.25 (Figure 7).

#### 3.1.3. Clinical and Clinicopathological Data and ESR Descriptive Statistics in the Subgroup of Anemic Cats

The CBC was performed on 111 cat samples, and anemia was detected in 43 (37/43, 86% non-regenerative). Anemia was mild in 39 cats and moderate in four cats. Thirty-two anemic cats (74%) had clinical signs and/or clinicopathological abnormalities compatible with systemic inflammation. Erythrocytosis was found in two cats.

**Figure 8:**
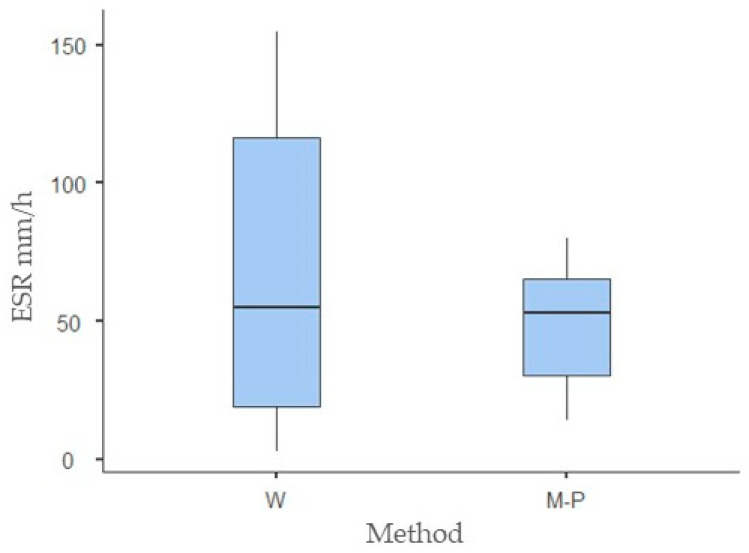
Box plot of ESR values obtained with the Westergren (W) method and MINI-PET (M-P) device in anemic cats.

No significant difference was observed in anemic cats between the ESR M-P and ESR W values (Table 3, Figure 8).

**Table 3:** Descriptive statistics of the ESR values (mm/h) obtained in anemic cats with the Westergren method (W) and MINI-PET (M-P) device.

	W	M-P
Number of values	43	43
Minimum	3.00	14.00
25th percentile	16.00	29.00
Median	55.00	53.00
75th percentile	117.00	66.00
Maximum	155.00	80.00

**Figure 9:**
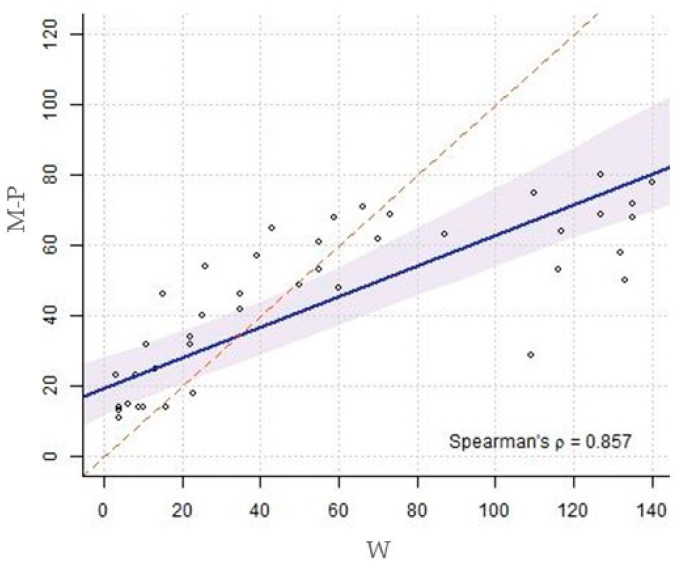
Passing–Bablok regression of ESR values (mm/h) obtained with the Westergren (W) method and MINI-PET (M-P) device in anemic cats.

A significant (*p* < 0.0001), positive, and very strong (r*_s_* = 0.86) correlation was detected between ESR M-P and ESR W values. The intercept of the Passing–Bablok regression analysis was estimated at 10.32 (95% CI: 9.00–13.00), and the slope was at 0.62 (95% CI: 0.54–0.71) (Figure 9).

**Figure 10:**
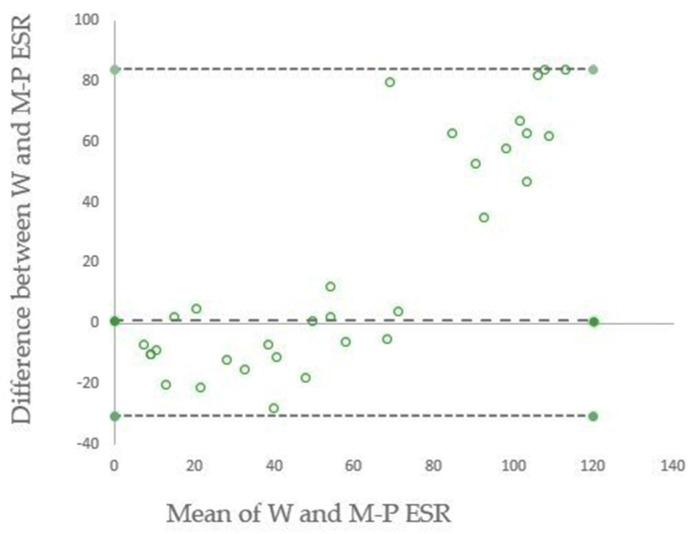
Bland–Altman plot of ESR values (mm/h) obtained with the Westergren (W) method and MINI-PET (M-P) device in anemic cats. Dashed line represents the bias (median of differences), and dotted lines indicate the upper and lower limits of agreement (2.5th and 97.5th percentiles).

The Bland–Altman analysis showed a bias of 1, with a 95% confidence interval ranging from −30.7 to 84 (Figure 10).

#### 3.1.4. Comparison of Westergren and MINI-PET ESR Values Between All Cats Examined, Cats with Erythrocyte Count, Hemoglobin, and Hematocrit Values Within Reference Range, and Anemic Cats

ESR values in anemic cats were significantly higher than in cats with erythrocyte counts, hemoglobin, and hematocrit values within reference ranges, as measured by both the Westergren method (*p* = 0.0080) and the MINI-PET assay (*p* = 0.0120) (Table 1, Table 2 and Table 3, Figure 2, Figure 5 and Figure 8). 

### 3.2. Dog Demographic Data

One hundred and forty-two dogs were included in this study. Sixty-three dogs (44.4%) were crossbreeds, 71 (50%) were purebreds, and the breed was not reported in eight dogs (5.6%). Males numbered 84 (59.2%), and females numbered 58 (40.8%). The age of 109 dogs ranged from 6 to 204 months (median: 60 months; 25th percentile: 36 months; 75th percentile: 96 months), and in 33 dogs, the age was unknown.

#### 3.2.1. Canine ESR Descriptive Statistics

**Figure 11:**
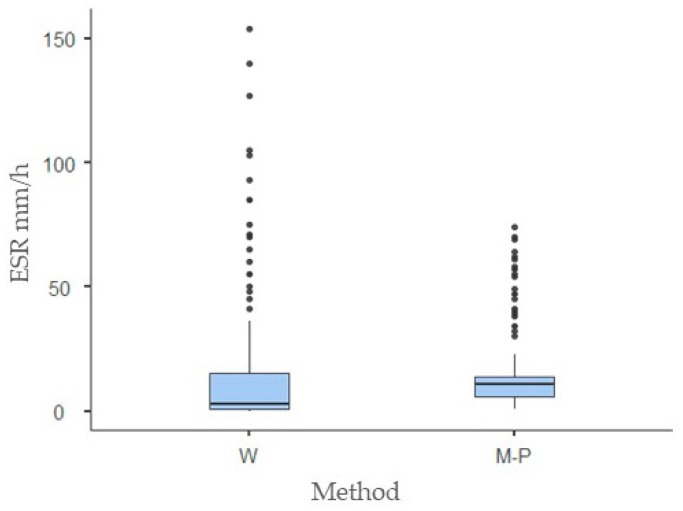
Box plot of ESR values obtained with the Westergren (W) method and MINI-PET (M-P) device in all dogs examined.

ESR M-P values were significantly higher than ESR W measures (*p* = 0.0013) (Table 4, Figure 11).

**Table 4:** Descriptive statistics concerning the ESR values (mm/h) obtained with the Westergren (W) method and MINI-PET (M-P) device in all dogs examined.

	W	M-P
Number of values	142	142
Minimum	0.00	1.00
25th percentile	1.00	6.00
Median	3.00	11.00
75th percentile	15.25	14.00
Maximum	154.00	74.00

A significant (*p* < 0.0001), positive, and strong correlation was detected between ESR M-P and ESR W values (r*_s_* = 0.75). 

**Figure 12:**
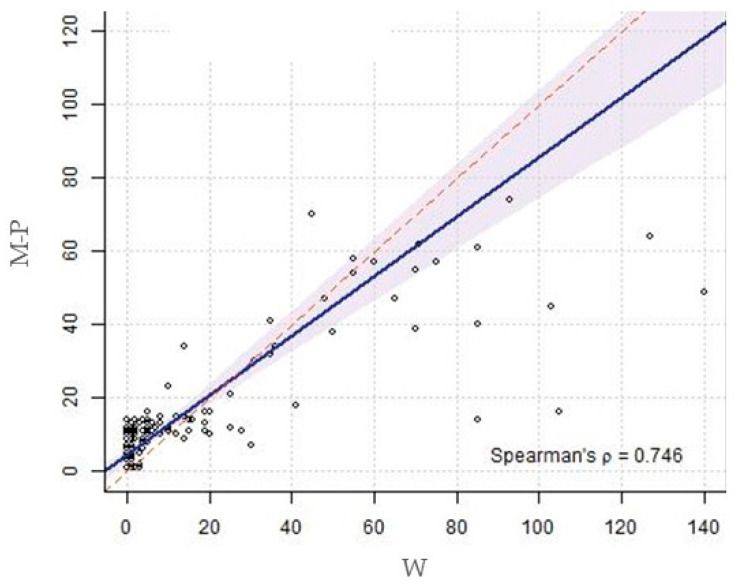
Passing–Bablok regression of ESR values obtained with the Westergren (W) method and MINI-PET (M-P) device in all dogs examined.

According to the Passing–Bablok regression analysis, the intercept was estimated at 4.58 (95% CI: 2.94–6.12), and the slope was at 0.81 (95% CI: 0.69–1.00) (Figure 12).

**Figure 13:**
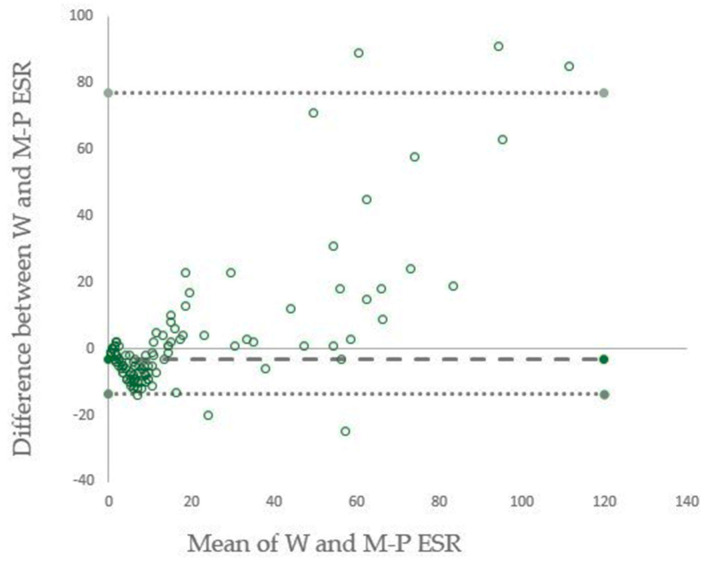
Bland–Altman plot of ESR values obtained with the Westergren (W) method and MINI-PET (M-P) device in all dogs examined. Dashed line represents the bias (median of differences), and dotted lines indicate the upper and lower limits of agreement (2.5th and 97.5th percentiles).

The Bland–Altman analysis showed a bias of −3, with a 95% confidence interval ranging from −13.43 to 76.95 (Figure 13). 

The following decision limits were established in healthy dogs after the exclusion of outliers (one ESR W value): ESR W: ≤3 mm/h and ESR M-P: ≤14 mm/h. 

#### 3.2.2. Canine ESR Descriptive Statistics in the Dog Subgroup with Erythrocyte Counts, Hemoglobin, and Hematocrit Values Within Reference Ranges

**Figure 14:**
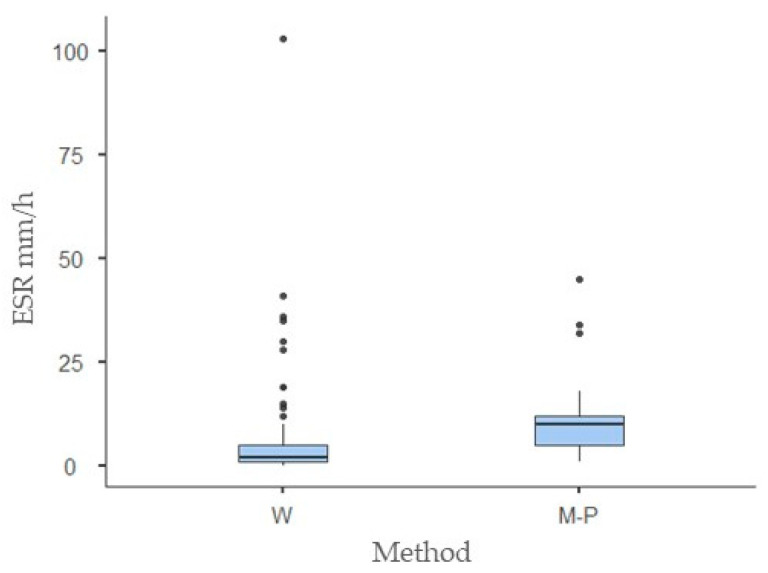
Box plot of ESR values obtained with the Westergren (W) method and MINI-PET (M-P) in dogs with erythrocyte count, hemoglobin, and hematocrit values within reference ranges.

In 70 dogs with erythrocyte counts, hemoglobin, and hematocrit values within reference ranges, the ESR M-P values were significantly higher than the ESR W measurements (*p* = 0.0001) (Table 5, Figure 14).

**Table 5:** Descriptive statistics of ESR values (mm/h) obtained with the Westergren (W) method and MINI-PET (M-P) device in dogs with erythrocyte counts, hemoglobin, and hematocrit values within reference ranges.

	W	M-P
Number of values	70	70
Minimum	0.00	1.00
25th percentile	1.00	5.00
Median	2.00	10.00
75th percentile	5.00	12.00
Maximum	103.00	45.00

**Figure 15:**
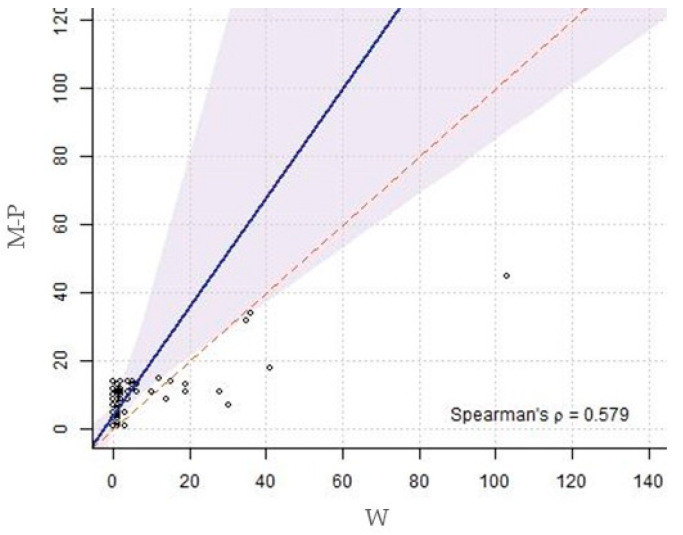
Passing–Bablok regression of ESR values obtained with the Westergren (W) method and MINI-PET (M-P) device in dogs with erythrocyte count, hemoglobin, and hematocrit values within reference ranges.

A significant (*p* < 0.0001), positive, and moderate (r*_s_* = 0.58) correlation was detected between ESR M-P and ESR W measures. According to the Passing–Bablok regression analysis, the intercept was estimated at 4.40 (95% CI: 1.00–7.00), and the slope was at 1.60 (95% CI: 0.78–3.52) (Figure 15).

**Figure 16:**
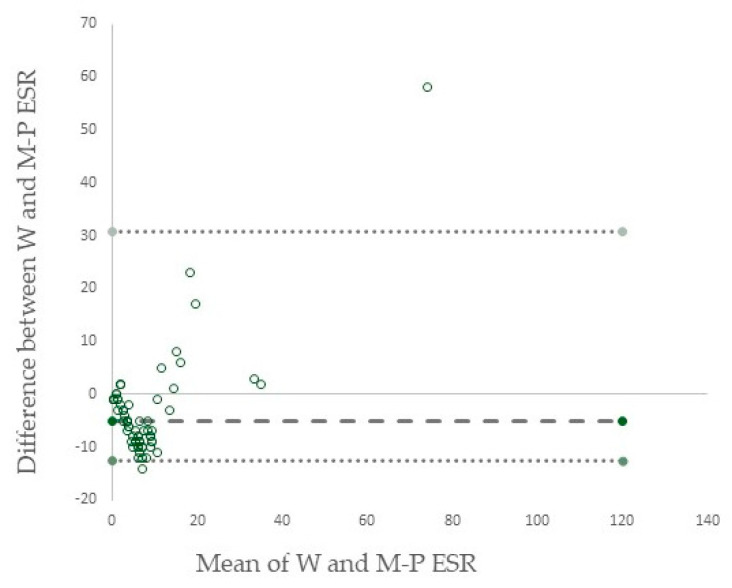
Bland–Altman plot of ESR values obtained with Westergren (W) method and MINI-PET (M-P) device in dogs with erythrocyte count, hemoglobin, and hematocrit values within reference ranges. Dashed line represents the bias (median of differences), and dotted lines indicate the upper and lower limits of agreement (2.5th and 97.5th percentiles).

The Bland–Altman analysis showed a bias of −5, with a 95% confidence interval ranging from −12.45 to 30.87 (Figure 16).

#### 3.2.3. Clinical and Clinicopathological Data and ESR Descriptive Statistics in the Subgroup of Anemic Dogs

The CBC was performed in 100 dogs, and anemia was detected in 24 (17/24, 71% non-regenerative). Anemia was mild in nine dogs, moderate in 11, and severe in four dogs. Almost all anemic dogs (23/24) had clinical signs and/or clinicopathological abnormalities compatible with systemic inflammation. Erythrocytosis was found in six dogs.

**Figure 17:**
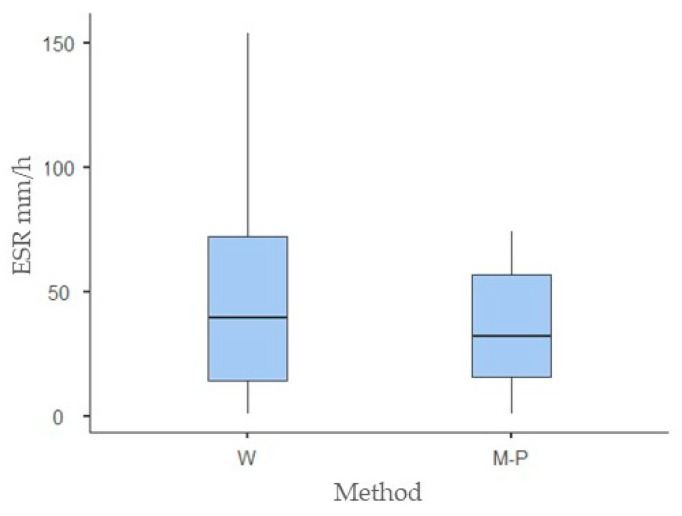
Box plot of ESR values obtained with the Westergren (W) method and MINI-PET (M-P) device in anemic dogs.

The ESR W values were significantly higher than ESR M-P measures in anemic dogs (*p* = 0.0358) (Table 6, Figure 17).

**Table 6:** Descriptive statistics of the ESR values (mm/h) obtained with the Westergren (W) method and MINI-PET (M-P) device in anemic dogs.

	W	M-P
Number of values	24	24
Minimum	1.00	1.00
25th percentile	14.00	15.25
Median	39.50	32.00
75th percentile	74.00	57.00
Maximum	154.00	74.00

**Figure 18:**
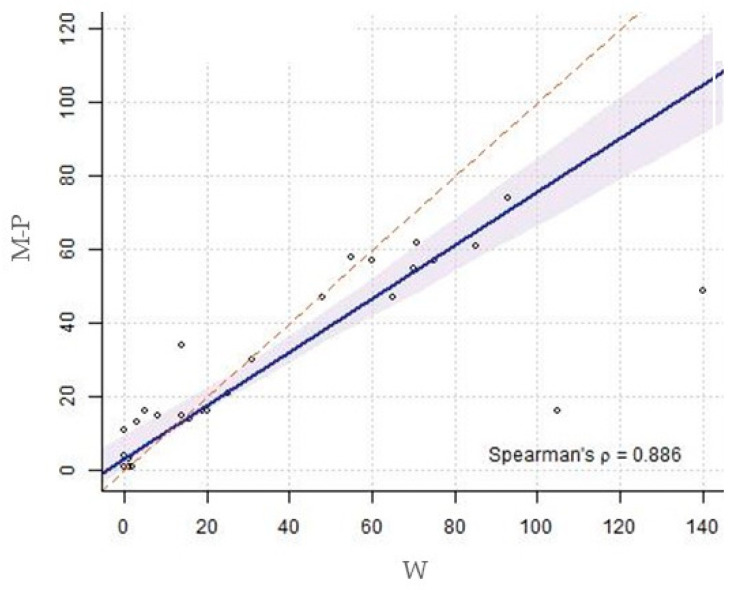
Passing–Bablok regression of ESR values obtained with the Westergren (W) method and MINI-PET (M-P) device in anemic dogs.

A significant (*p* < 0.0001), positive, and very strong (r*_s_* = 0.89) correlation was detected between ESR M-P and ESR W values. According to the Passing–Bablok regression analysis, the intercept was estimated at 3.46 (95% CI: 1.00–10.03), and the slope was at 0.72 (95% CI: 0.60–0.84) (Figure 18). 

**Figure 19:**
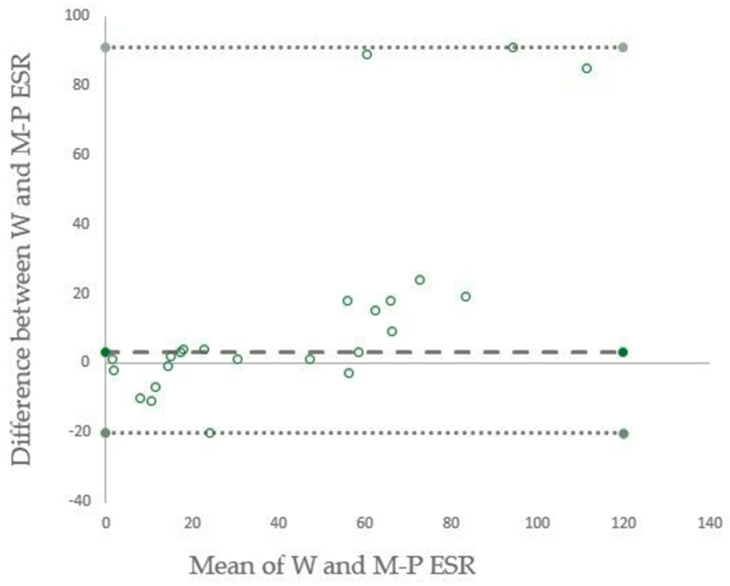
Bland–Altman plot of ESR values obtained with the Westergren (W) method and MINI-PET (M-P) device in anemic dogs. Dashed line represents the bias (median of differences), and dotted lines indicate the upper and lower limits of agreement (2.5th and 97.5th percentiles).

The Bland–Altman analysis showed a bias of 3, with a 95% confidence interval ranging from −20 to 91 (Figure 19).

#### 3.2.4. Comparison of ESR Values from Westergren and MINI-PET Between All Dogs Examined, Dogs with Normal Erythrocyte Count, Hemoglobin, and Hematocrit Values, and Anemic Dogs

ESR values in anemic dogs were significantly higher than in dogs with erythrocyte counts, hemoglobin, and hematocrit values within reference ranges (*p* < 0.0001), and they were higher than in all dogs examined (*p* < 0.0001), as measured by both methods (Table 4, Table 5 and Table 6, Figure 11, Figure 14 and Figure 17).

## 4. Discussion

### 4.1. Discrepancy Among ESR Values Measured with MINI-PET and Westergren Methods

A very strong (overall cats) or strong (overall dogs) positive correlation among ESR values measured with MINI-PET and Westergren methods was found, as previously seen in horses and cats [17,23]. Conversely, the analytical agreement was low in both species and all groups. Passing–Bablok analyses revealed significant constant and proportional biases, and Bland–Altman plots showed wide limits of agreement, especially at higher ESR values (Figure 3, Figure 4, Figure 6, Figure 7, Figure 9, Figure 10, Figure 12, Figure 13, Figure 15, Figure 16, Figure 18 and Figure 19). These findings show that, in the clinically heterogeneous feline and canine population studied, the two methods did not yield interchangeable measures across the entire measurement range. Because of the lack of analytical agreement between the two methods, the ESR report must always state the methodology used, and in the case of ESR monitoring, the same method should be used for serial measurements. No published data are available about the agreement between these two ESR methods in dogs. In cats, Gori et al. (2024) found, in 60 healthy and sick cats, a lower Bland–Altman bias value (−0.1) and narrower confidence interval range (−21.8, +21.6) compared to the present study (bias value, −4.5; 95% CI range: −29, +83.6) [23]. In fact, they found a maximum mean Westergren/MINI-PET value of 80 mm/h, while we had a value as high as 120 mm/h (Figure 4) [23]. 

Unlike most laboratory tests, the ESR does not measure a well-defined analyte with a specific molecular structure but rather a physicochemical phenomenon, and true standardization of new ESR technologies is, by definition, impossible [24]. There are many different automated ESR systems used in human medicine, and they do not measure the same pathophysiological processes as the Westergren method [24]. They rely on shorter detection times and use blood with anticoagulants other than Westergren and different blood sample dilutions, as emphasized by the ICSH [24]. In humans, it is well known that modified Westergren methods that have, such as the MINI-PET device, sedimentation times of less than 60 min and use mathematical extrapolations to one hour may show significant differences compared to the manual Westergren method, particularly at higher values, as observed in the case of dogs and cats with ESR levels >60–80 mm/h (Figure 4, Figure 7, Figure 10, Figure 13, Figure 16 and Figure 19) [24]. In fact, while the cut-off values of disease were lower with the Westergren method (cat ≤ 15 mm/h; dog ≤ 3 mm/h) compared to MINI-PET (cat ≤ 25 mm/h; dog ≤ 14 mm/h), the ESR W measurements reached maximum values about twice as high as the ESR M-P values in both cats (Table 1) and dogs (Table 4).

### 4.2. ESR Values in Anemic Cats and Dogs

Anemic cats and dogs had higher ESR values than the matched subgroups of animals with erythrogram values within the reference range using both methods. A negative correlation between ESR values and hematocrit is well known in humans [4]. In dogs with various diseases, a negative correlation was found among ESR M-P values and hematocrit [20,21,31], and in cats with chronic kidney disease, it was detected between both hematocrit and hemoglobin measures [11]. 

Based on clinical and clinicopathological data evaluated in anemic cats and dogs, an inflammatory condition was probably associated with anemia in most of these animals. We cannot therefore exclude that inflammation, which usually leads to mild-to-moderate non-regenerative anemia, contributed to the ESR increase in anemic animals and possibly to anemia as well. Nevertheless, the erythrogram evaluation of dogs and cats tested with ESR is recommended. In the case of serial ESR evaluations, changes in erythrogram and ESR values should be evaluated in conjunction to correctly interpret any changes in ESR measures. The MINI-PET method is a better option in these cases to save the extra 1 mL of blood needed for the Westergren method, particularly in cases of anemia, puppies, kittens, cats, and small-breed dogs.

### 4.3. Healthy Cats Have Higher ESR Values Compared to Healthy Dogs

As reported in other studies, we found significantly higher ESR values in healthy cats than in healthy dogs regardless of the method used [11,18,22,23,31]. The higher decision limits in cats compared to dogs can be explained by the natural tendency of feline RBCs to form rouleaux under physiological conditions. This physical phenomenon promotes sedimentation and is probably the main reason for the higher ESR measures compared to healthy dogs [32]. However, the maximum ESR M-P values measured were comparable across dogs and cats (Table 1 and Table 4). 

The ESR M-P decision limits (cat ≤ 25 mm/h in cats; dog ≤ 14 mm/h) were higher than ESR W (cat ≤ 15 mm/h; dog ≤ 3 mm/h) in both species. Few studies provided feline and canine MINI-PET reference intervals according to ASVCP guidelines [30]. Gori et al. (2023, 2024) calculated 1–23 mm/h and 1–8 mm/h as their cat and dog reference intervals, respectively [18,23]. Paltrinieri et al. (2024) and Joo et al. (2026), respectively, reported median values of 7 (IQR 2.7–10) mm/h and 10 (IQR: 4–11) mm/h in healthy dogs [22,31]. These values are similar to the descriptive statistic values of our healthy cats and dogs (Supplementary File S1: Tables S1 and S2).

### 4.4. Limitations of This Study and Future Perspectives

The main limitation of our study arises from both the cat and dog sample sizes. This limitation precluded the possibility to calculate reference limits according to AVSCP guidelines for reference intervals. We established decision limits, i.e., an ESR value threshold that we assumed to differentiate between healthy and non-healthy individuals in the dogs and cats studied. 

Using two different laser veterinary CBC analyzers is, in general, a methodological approach of a CBC assessment study. However, the comparison of the diagnostic performance of the two blood cell counters performed previously did not report significant discrepancies in the erythrogram parameters evaluated in anemic animals.

An additional limitation is the generic clinical classification of investigated animals, apart from the diagnosis of anemia. The heterogeneity of the population studied could be responsible for the highly dispersed values detected in all groups. Paltrineri et al. (2024) aimed to assess the conditions in which the ESR increases more frequently in canine practice [31]. They reported the highest ESR values and a higher frequency of elevated ESR measures in dogs with acute/subacute inflammation and in the chronic kidney disease dog group; however, within all their dog disease groups, ESR M-P values were highly dispersed [31]. Finally, the anemic subgroups did not include an adequate number of animals with severe anemia and animals with anemia not associated with inflammation. 

Reference intervals based on large studies and investigations of physiological factors that may interfere with ESR M-P values (e.g., sex, reproductive status, age, breed) are warranted, as well as performance metrics of the test under different inflammatory and non-inflammatory conditions, in order to validate the clinical use of ESR in dogs and cats. A further field of study is the kinetics of ESR M-P values during remission of the inflammatory process, required for the clinical interpretation of follow-up data.

## 5. Conclusions

The ESR M-P and ESR W methods have different decision limits in the studied dogs and cats, and a method-specific interpretation of their results is suggested. Cats have higher ESR decision limits compared to dogs. Anemia was associated with higher ESR values, probably due to concurrent inflammation in most anemic animals studied; however, ESR measures should be evaluated in conjunction with erythrogram values. Due to the limitations caused by sample sizes and heterogeneity, these conclusions should be interpreted with caution. 

## Data Availability

The raw data supporting the conclusions of this article will be made available by the authors upon request.

## Supplementary Materials

The following supporting information can be downloaded at: https://www.mdpi.com/article/10.3390/ani16152298/s1, Table S1: ESR values (mm/h) measured in the healthy cats selected to calculate decision limits (Westergren: W; MINI-PET: M-P), and the respective number of observations per individual value (n.). W mean (standard deviation) = 5.7 (4.2) mm/h; M-P mean (standard deviation) = 12.7 (5.4) mm/h. Table S2: ESR values (mm/h) measured in the healthy dogs selected to calculate decision limits (Westergren: W; MINI-PET: M-P), and the respective number of observations per individual value (n.). W-mean (standard deviation) = 1.1 (0.8) mm/h; M-P median (25th–75th percentiles) = 9 (1-11) mm/h. Figure S1. Histogram of ESR values measured in healthy cats selected to calculate decision limits (Westergren: W; MINI-PET: M-P). Figure S2. Histogram of ESR values measured in healthy dogs selected to calculate decision limits (Westergren: W; MINI-PET: M-P).

## Abbreviations

The following abbreviations are used in this manuscript:

ALP	Alkaline phosphatase
ALT	Alanine aminotransferase
BUN	Blood urea nitrogen
CBC	Cell blood count
CI	Confidence interval
EDTA	Ethylenediaminetetraacetic acid
ESR	Erythrocyte sedimentation rate
ESR M-P	ESR measures of MINIPET
ESR W	ESR measures of Westergren
HCT	Hematocrit
ICSH	International Council for Standardization in Hematology
M-P	MINI-PET
NC	Not calculable
NPV	Negative predictive value
POC	Point of care
PPV	Positive predictive value
RBC	Red blood cell
TATs	Turn-around times
W	Westergren

## References

[1] Westergreen A.V.A. (1921). Studies of the suspension stability of the blood in pulmonary tuberculosis. Acta Med. Scand..

[2] Jou J.M., Lewis S.M., Briggs C., Lee S.H., De La Salle B., McFadden S. (2011). International Council for Standardization in Haematology ICSH review of the measurement of the erythocyte sedimentation rate. Int. J. Lab. Hematol..

[3] Darras A., Breunig H.G., John T., Zhao R., Koch J., Kummerow C., König K., Wagner C., Kaestner L. (2022). Imaging erythrocyte sedimentation in whole blood. Front. Physiol..

[4] Diamanti D., Pieroni C., Pennisi M.G., Marchetti V., Gori E., Paltrinieri S., Lubas G. (2025). The erythrocyte sedimentation rate (ESR) in veterinary medicine: A focused review in dogs and cats. Animals.

[5] Jain N.C., Kono C.S. (1975). Erythrocyte sedimentation rate in the dog and cat: Comparison of two methods and influence of packed cell volume, temperature and storage of blood. J. Small Anim. Prac..

[6] Fabry T.L. (1987). Mechanism of erythrocyte aggregation and sedimentation. Blood.

[7] Talstad I., Haugen H.F. (1979). The relationship between the erythrocyte sedimentation rate (ESR) and plasma proteins in clinical materials and models. Scand. J. Clin. Lab. Investig..

[8] Allen B.V. (1988). Relationships between the erythrocyte sedimentation rate, plasma proteins and viscosity, and leucocyte counts in thoroughbred racehorses. Vet. Rec..

[9] Narang V., Grover S., Kang A.K., Garg A., Sood N. (2020). Comparative analysis of erythrocyte sedimentation rate measured by automated and manual methods in anaemic patients. J. Lab. Physicians.

[10] Kahar M.A. (2022). Erythrocyte sedimentation rate (with its inherent limitations) remains a useful investigation in contemporary clinical practice. Ann. Pathol. Lab. Med..

[11] Uva A., Cavalera M.A., Gusatoaia O., Donghia R., Gernone F., Silvestrino M., Zatelli A. (2023). Inflammatory status and chronic kidney disease in cats: Old and new inflammatory markers-a pilot prospective study. Animals.

[12] Donato G., Caspanello T., Caprì A., De Majo M., Iannelli N.M., Rosace F., Bruno F., Castelli G., Pennisi M.G., Masucci M. (2024). The erythrocyte sedimentation rate and other markers of inflammation in cats tested for *Leishmania infantum* and feline immunodeficiency virus antibodies. Parasites Vectors.

[13] Johnson V., Burgess B., Morley P., Bragg R., Avery A., Dow S. (2016). Comparison of cytokine responses between dogs with sepsis and dogs with immune-mediated hemolytic anemia. Vet. Immunol. Immunopathol..

[14] Tasker S., Hofmann-Lehmann R., Belák S., Frymus T., Addie D.D., Pennisi M.G., Boucraut-Baralon C., Egberink H., Hartmann K., Hosie M.J. (2018). Haemoplasmosis in cats: European guidelines from the ABCD on prevention and management. J. Feline Med. Surg..

[15] von Hohnhorst I.M., Moritz A., Eisenecker C.M., Strube C., Rodjana K.E., Müller E., Schäfer I. (2025). Impact of levels of parasitemia and antibodies, acute-phase proteins, as well as stays abroad on hematological and biochemical parameters in 342 dogs with acute *Babesia canis* infection. Parasites Vectors.

[16] Horton S., Fleming K.A., Kuti M., Looi L.M., Pai S.A., Sayed S., Wilson M.L. (2019). The top 25 laboratory tests by volume and revenue in five different countries. Am. J. Clin. Pathol..

[17] Pieroni C., Grassi A., Pantoli M., Berretti M., Messina S., Giovannini C., Lubas G., Diamanti D. (2023). Analytical validation of MINI-PET as point-of-care for erythrocyte sedimentation rate measure in horses. Vet. Med. Int..

[18] Gori E., Pierini A., Pasquini A., Diamanti D., Carletti C., Lubas G., Marchetti V. (2023). The erythrocyte sedimentation rate (ESR) in canine inflammation. Vet. J..

[19] Cavalera M.A., Gusatoaia O., Uva A., Gernone F., Tarallo V.D., Donghia R., Silvestrino M., Zatelli A. (2024). Erythrocyte sedimentation rate in heartworm naturally infected dogs “with or without” *Leishmania infantum* seropositivity: An observational prospective stud. Front. Vet. Sci..

[20] Cavalera M.A., Gernone F., Uva A., Donghia R., Carelli G., Iatta R., Zatelli A. (2022). Erythrocyte sedimentation rate in canine leishmaniosis diagnosis: A new resource. Front. Vet. Sci..

[21] Lubas G., Paltrinieri S., Papini R.A., Lensi I., Benali S.L., Cortadellas O., D’Anna N., Fondati A., Roura X., Zini E. (2024). Clinical and clinico-pathological observations of the erythrocyte sedimentation rate in dogs affected by leishmaniosis and other inflammatory diseases. Animals.

[22] Joo J.B., Kim K., Ro W.B., Lee C.M. (2026). The Erythrocyte Sedimentation Rate as a Novel Prognostic Marker in Canine Inflammatory Diseases. Animals.

[23] Gori E., Pasquini A., Paltrinieri S., Lubas G., Militello C., Diamanti D., Carletti C., Pantoli M., Marchetti V. (2024). Prospective application of the erythrocyte sedimentation rate (ESR) as a possible inflammatory marker in feline patients. Vet. Med. Int..

[24] Kratz A., Plebani M., Peng M., Lee Y.K., McCafferty R., Machin S.J., International Council for Standardization in Haematology (ICSH) (2017). ICSH recommendations for modified and alternate methods measuring the erythrocyte sedimentation rate. Int. J. Lab. Hematol..

[25] ICSH (1993). ICSH recommendations for measurement of erythrocyte sedimentation rate. International Council for Standardization in Haematology (Expert Panel on Blood Rheology). J. Clin. Pathol..

[26] Tvedten H., Weiss D.J., Wardrop K.J. (2010). Laboratory and clinical diagnosis of anemia. Schalm’s Veterinary Hematology.

[27] Ramsey P.H. (1989). Critical values for Spearman’s rank order correlation. J. Educ. Stat..

[28] The BMJ Correlation and Regression. https://bmj-chicken.bmj.com/thebmj/about-bmj/resources-readers/publications/statistics-square-one/11-correlation-and-regression.

[29] Vidali M., Tronchin M., Dittadi R. (2016). Protocollo per la comparazione di due metodi analitici di laboratorio. Biochim. Clin..

[30] Friedrichs K.R., Harr K.E., Freeman K.P., Szladovits B., Walton R.M., Barnahart K.F., Blanco-Chavez J. (2012). ASVCP reference interval guidelines: Determination of de novo reference intervals in veterinary species and other related topics. Vet. Clin. Pathol..

[31] Paltrinieri S., Ferrari R., Scavone D., Pieroni C., Diamanti D., Tagliasacchi F. (2024). Increased erythrocyte sedimentation rate in dogs: Frequency in routine clinical practice and association with hematological changes. Animals.

[32] Harvey J.W. (2012). Evaluation of erythrocyes. Veterinary Hematology: A Diagnostic Guide and Color Atlas.

