# Supplementary material for: Comparison of Westergren and Automated Erythrocyte Sedimentation Rate Measurements in Dogs and Cats
Source: Animals (Basel). 2026 Jul 24;16(15):2298. doi: 10.3390/ani16152298 (PMC13463589; doi:10.3390/ani16152298)

Table S1: ESR values (mm/h) measured in the healthy cats selected to calculate decision limits (Westergren: W; MINI-PET: M-P), and the respective number of observations per individual value (n.). W mean (standard deviation) = 5.7 (4.2) mm/h; M-P mean (standard deviation) = 12.7 (5.4) mm/h.

W	n.	M-P	n.
0	1	1	2
1	2	5	1
3	2	9	1
4	3	11	2
8	1	12	1
9	1	13	2
10	1	14	8
11	1	15	3
15	1	21	1
		25	1

Figure S1. Histogram of ESR values measured in healthy cats selected to calculate decision limits (Westergren: W; MINI-PET: M-P).

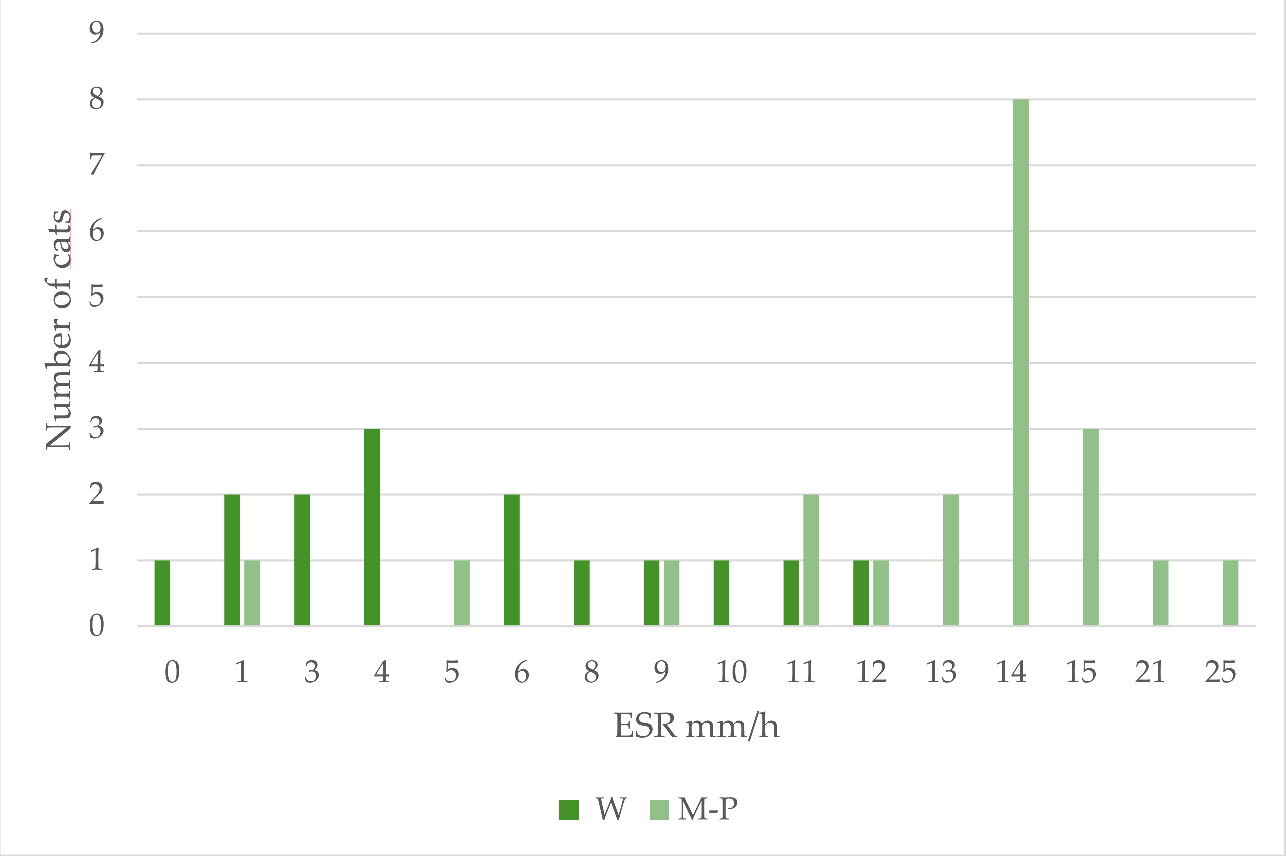

Table S2: ESR values (mm/h) measured in the healthy dogs selected to calculate decision limits (Westergren: W; MINI-PET: M-P), and the respective number of observations per individual value (n.). W-mean (standard deviation) = 1.1 (0.8) mm/h; M-P median (25th – 75th percentiles) = 9 (1-11) mm/h.

W	n.	M-P	n.
0	7	1	8
1	11	3	1
2	5	4	2
3	1	7	1
		9	2
		10	2
		11	6
		12	1
		13	1
		14	1

Figure S2. Histogram of ESR values measured in healthy dogs selected to calculate decision limits (Westergren: W; MINI-PET: M-P).

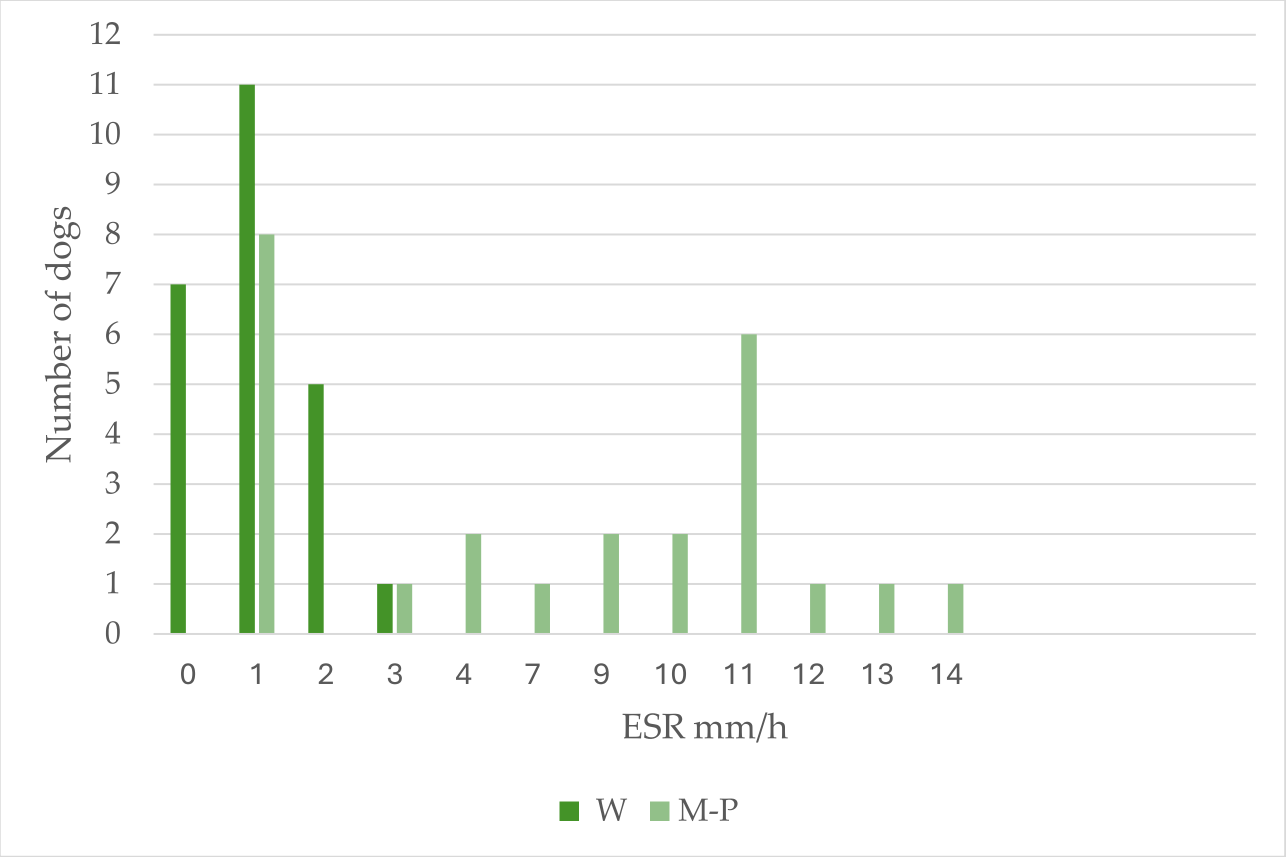

Supplement: Supplementary file 1 [file animals-16-02298-s001.zip › animals-4382936-supplementary.pdf]
